# Galvanic vestibular stimulation with low intensity improves dynamic balance

**DOI:** 10.1515/tnsci-2020-0197

**Published:** 2021-12-06

**Authors:** Hongmei Chen, Zhen Hu, Yujuan Chai, Enxiang Tao, Kai Chen, Tetsuya Asakawa

**Affiliations:** School of Mechanical Engineering, Hangzhou Dianzi University, No. 1158, Xiasha 2nd Street, Jianggan District, Hangzhou, Zhejiang 310018, China; Department of Neurology, Ruijin Hospital Affiliated to Shanghai Jiao Tong University, Shanghai 200000, China; School of Medical Engineering, Health Science Center, Shenzhen University, Shenzhen 518060, China; Department of Neurology, The Eighth Affiliated Hospital, Sun Yat-Sen University, Shenzhen 518033, China; Research Base of Traditional Chinese Medicine Syndrome, Fujian University of Traditional Chinese Medicine, Fuzhou 350122, China

**Keywords:** dynamic balance, galvanic vestibular stimulation with very low intensity direct current, rambling, trembling, digital controlled rocker force platform, fall risk

## Abstract

**Background:**

Dynamic balance is associated with fall risk. The aim of this study is to explore the effects of galvanic vestibular stimulation with very low intensity direct current (dcGVS) on dynamic balance.

**Methodology:**

We used a rocker force platform for assessing the dynamic balance performance. Center-of-pressure (COP) coordinates were acquired and decomposed to rambling (RA) and trembling (TR). We measured sway parameters, including length, average speed, and average range, affected by dcGVS at 0.01 mA with eyes open (EO) and eyes closed (EC).

**Results:**

We assessed 33 young healthy subjects and found that all sway parameters were shorter in the EO condition, indicating a better dynamic balance performance. dcGVS significantly improved the dynamic balance performance both in EO and EC conditions. All the sway parameters in COP in EO were significantly shorter than those in EC, indicating a better dynamic balance performance in EO. In EO, RA had greater improvement rates than TR. In EC, only average speed had a greater improvement rate in RA, whereas length and average range had greater improvement rates in TR. These results indicate a different modulation model between EO and EC.

**Conclusion:**

These findings indicate that very low intensity dcGVS improved the sway parameters of dynamic balance in young healthy subjects. Moreover, our results suggest different dynamic balance control models between having EO and EC. The mechanisms of these phenomena caused by very low intensity dcGVS require further investigation.

## 1 Introduction

Balance is an element of physical fitness that resists external forces and maintains body stability. It is an essential element of daily living. Balance includes static balance (an undisturbed condition) and dynamic balance (a response to internal or external disturbances) [1]. Investigation of the mechanisms of balance has been of increasing interest to researchers because disturbances in balance increase fall risk, which can impede a patient’s daily activities and quality of life. The mechanisms underlying balance and balance dysfunction are complicated and not fully clear. Body balance is mainly modulated by the vestibular system. The three semicircular canals of the vestibular system are associated with the speed and angular acceleration of the head, whereas the otolithic organ senses the head’s linear acceleration [2].

Dysfunction of the vestibular system results in balance disturbance and increased fall risk. However, therapeutics against vestibular dysfunctions are limited. Besides conventional physical therapy, noisy galvanic vestibular stimulation (nGVS) is reported to help improve gait parameters in patients with vestibular dysfunction [3]. Moore et al. reported that nGVS contributes to the enhancement of the sensorimotor performance in novel vestibular environments [4]. Inukai et al. found that nGVS contributes to improving postural sway in an open-eye standing posture among young subjects [5]. Iwasaki et al. reported that nGVS decreases center-of-pressure (COP) velocity and sway area under static balance in healthy adults [6]. nGVS is also considered to be an acceptable therapy for vestibular dysfunction. A recent study by Chen et al. found that nGVS remarkably reduced walking deviations, particularly in a visual-deprived state in patients with bilateral vestibular hypofunction (BVH). Using nGVS under different conditions (head rotation frequency and light exposure level) may benefit to the rehabilitation of patients with BVH [7].

Different from nGVS, GVS with direct current (DC) (dcGVS) is a type of stimulation affecting the vestibular system [8]. It involves placing anodal and cathodal electrodes over the left and right mastoid and applying stimulation with low intensity current. The commonly used intensity is from 0.5 to 1.5 mA. Such stimulation may cause body sway in a person who is standing. Additionally, subjects undergoing dcGVS may feel the illusion that their heads are moving, and their whole bodies respond to the perceived head movement [9]. Due to the noninvasive and simple nature of dcGVS, it has been commonly used as a research tool for exploring the mechanisms of balance associated with the vestibular system. Earlier dcGVS studies focused on subjects who were standing still. Most of the earlier studies reported that stimulation of the unpredictable current waveform may achieve better postural stability [10] and postural performance [11]. Moore et al. found that the unpredictable GVS could ameliorate the spatial disorientation in pilots after spaceflight [12]. MacDougall pointed out that this improvement of postural stability can be attributed to amelioration of the vestibular function [10]. Samoudi et al. performed unpredictable GVS in patients with Parkinson’s disease (PD). They found that stimulation with current on 1 Hz frequency and 0.500 ± 0.255 mA (ranging between 0.1 and 0.9 mA) intensity improved the postural responses. They concluded that such stimulation in short term is safe and may have a small positive effect on amelioration of the motor symptoms in PD patients [13]. It has rarely been used to investigate the effects of constant-current GVS on dynamic balance, which is closely associated with fall risk [14]. MacDougall and his colleagues reported that long duration (more than 30 s) GVS at currents up to 5 mA evokes ocular torsion [15] and vestibular dysfunction [16]. However, no study directly involved in the observation of the effects of constant-current GVS on dynamic balance.

Assessments of dynamic balance have become crucial, particularly in patients with neurological disorders such as stroke [17] and PD [18]. Currently, most dynamic balance assessments are objective, such as single-leg jump landings [19], lose balance forward [20], and displacements and rotations of the support platform [21]. With the development of computerized technology, the development of a next-generation tool to achieve more precise behavioral assessment has been possible [22]. As in our earlier studies, we used the principle of objectification, multipurpose, and simplification to develop a novel behavioral assessment [18,22,23,24]. Accordingly, we modified a conventional digital-controlled rocker force platform (DCRFP) for assessing the dynamic balance performance [25]. As early as 1999, Zatsiorsky and Duarte developed a method to analyze COP trajectory [26]. The COP coordinates are decomposed and analyzed in terms of rambling (RA, represents a slow nonoscillatory component) and trembling (TR, represents a faster damped-oscillatory component) based on the concept of zero-force points or instant equilibrium points (IEPs). RA represents the complicated high central nervous system (CNS) response, and TR represents the elementary reflex, in which only spinal and muscular are involved [26]. It has been a canonical method to explore the postural sway mechanisms. In a typical RA–TR study, decomposition was conducted separately for the anterior–posterior (A/P) direction (*x* direction) and medial–lateral (M/L) direction (*y* direction). Trajectory of the slow component (RA) was commonly first separated from the COP trajectory when the horizontal forces reach zero, and then the trajectory of the fast component (TR) was calculated as the deviation of COP from the trajectory of RA. Using this method, the postural control function in different populations (such as in artistic gymnasts [27], in patients with PD [13,28], and in subjects with neck pain [29]) was investigated. Moreover, by using the DCRFP system and the Zatsiorsky analysis method, investigation of the effects of dcGVS on dynamic balance is possible. Our current study used this experimental system to investigate the effects of white Gaussian noise on dynamic balance in healthy young adults [25].

Based on the above knowledge, we designed this explorative study to observe the effects of dcGVS in a very low intensity. We observed these effects in young healthy adults during eyes open (EO) and eyes closed (EC) using the DCRFP system and the Zatsiorsky analysis method. This could contribute to a better understanding of the mechanisms of balance modulation.

## 2 Methods

### 2.1 Participants

A total of 33 healthy participants (average age 23.8 ± 2.0 years, range 21–25 years; 23 men and 10 women) were enrolled in this study. All participants underwent physical examination to confirm they were in a healthy state and did not have a history of postural or vestibular deficits.


**Informed consent:** Informed consent has been obtained from all individuals included in this study.
**Ethical approval:** The research related to human use has been complied with all the relevant national regulations, institutional policies, and in accordance with the tenets of the Helsinki Declaration of the World Medical Association (2000) and has been approved and supervised by the ethical committee of Shanghai Ruijin Hospital Luwan Branch (approval No: LWEC2019017).

### 2.2 dcGVS

dcGVS was performed using a DSP-305CM stimulator (Hongsheng Electronics Co., Shenzhen, China). A crescent-shaped electrode (2.0 cm in diameter) was used as the stimulating electrode. The stimulation locations were bilateral mastoid process [30]. Here, we used continuous stimulation with DC at a very low intensity. The selection of an appropriate stimulation intensity was extremely important in the present study. In the experiments performing unpredictable GVS, the current intensity was larger; for example, Samoudi et al. used an average intensity of 0.5 mA (0.1–0.9 mA, 1 Hz) for patients with PD; however, this stimulation was not a constant-current stimulation [13]. Reports using a constant-current GVS are limited. In earlier studies using dcGVS to investigate human vestibular responses, intensities ranged from 0.5 to 1.5 mA and caused notable compensatory movements of the head and body, and stimulation at more than 1 mA caused body rotation [31]. Studies by MacDougall et al. documented that a long-duration stimulation in constant current (≤5 mA) caused ocular torsion and vestibular dysfunction [15,16]. In Watson’s study, the ocular torsion occurred from stimulation from 1 mA [15]. In our preliminary study, we found that stimulation more than 0.4 mA caused discomfort in most of the participants. We, hence, considered that humans might be more sensitive to the long-duration stimulation in constant current. In this regard, we started stimulation from 0 mA and added the intensity with a very small intensity (0.01 mA). We found that the stimulation at 0.01 mA could evoke a significant stimulation effect but did not cause discomfort or any body movements. We, thus, set the current intensity at 0.01 mA.

### 2.3 Experimental procedures

**Figure 1:**
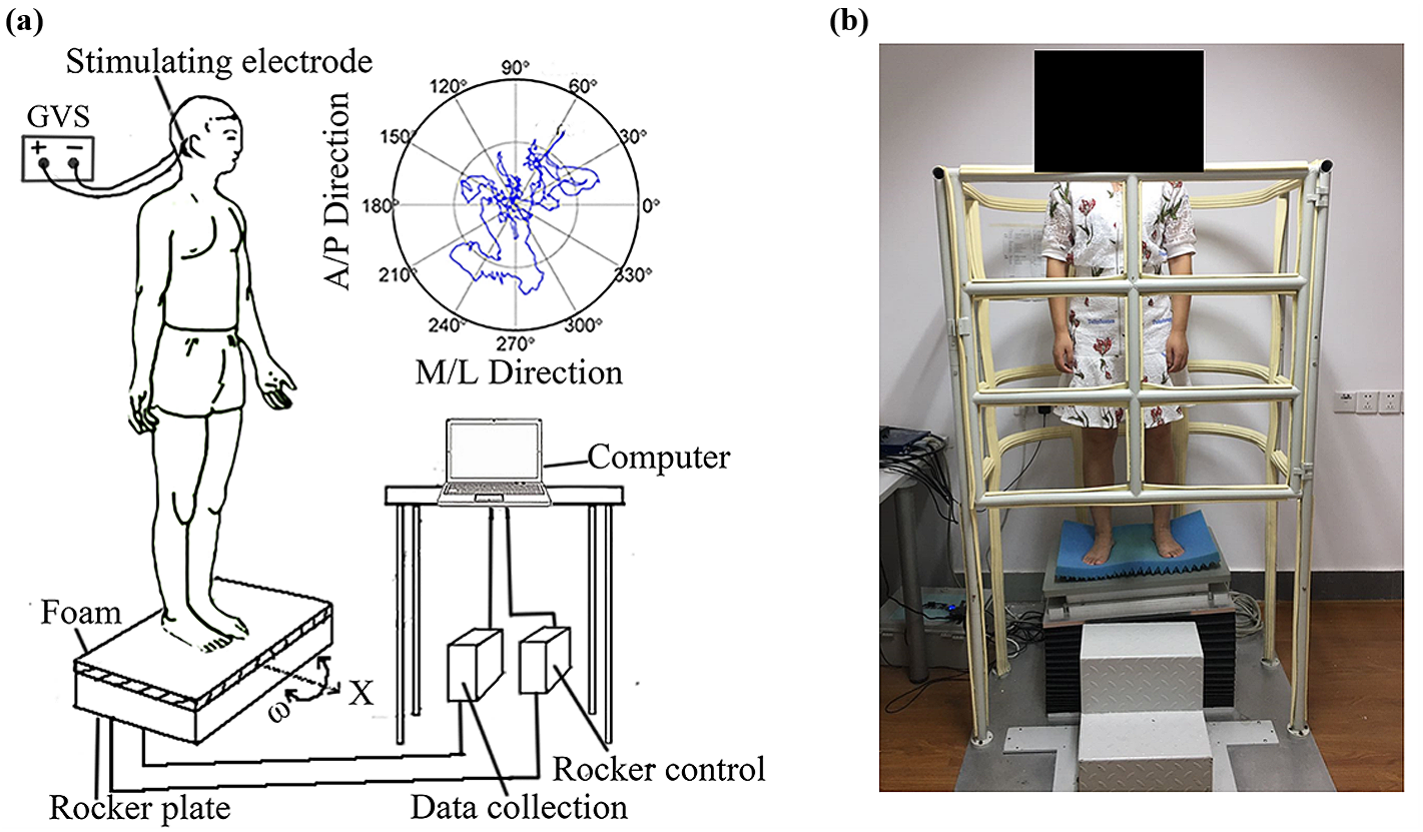
Experimental system of the novel DCRFP: (a) schematic of the DCRFP. The postural sway measures were performed with the dcGVS on or off and (b) photo taken during the experiments.

We modified a DCRFP system for assessing the dynamic balance performance based on our earlier research [25]. Briefly, our system consisted of an Advanced Mechanical Technology Inc. (AMTI) force plate (Model BP400600, Advanced Mechanical Technology Inc., MA, USA), a data collector, a rocker control, and a computer (Figure 1a). The platform includes the following parts: (1) a force plate (BP400600; Advanced Mechanical Technology Inc., MA, USA), (2) a swaying plate with 600 mm width and 400 mm length, (3) a swaying mechanism assembly, and (4) a safety ring. We set up the platform as our earlier study [25]. Briefly, the sway amplitude is set up by the angle *θ* (a sharp angle between the flat plate and the horizontal line), which is controlled by the servo motor motion control system, namely computer + servo motor + force platform (Figure 1a). During the experiments, computer controls servo drive to run the motor and then controls the parameters of the force platform (angle *θ* and frequency of sway). Once the rotation angle *θ* is determined, the COP coordinates can be measured through multiplication of a transformation matrix [32]. In the present study, angle *θ* was ±4° and frequency of sway was 1 Hz [25]. The COP coordinates were obtained by processing the feedback of rotation measurement and coordinate transformation from force plate data. All the experiments were performed in a quiet environment. We reset the device to zero each time before a participant stepped onto the platform, which was covered with a piece of 2 cm thick foam. This foam was set up to reduce the stimuli of somatosensory input and improve the specificity of the present study [33].

Each participant was instructed to stand barefoot in a comfortable foot position where was marked to ensure the feet locations of participants were exactly the same across trials. Before recording, each participant was asked to keep standing for 1 min to confirm the COPs were maintained in a relatively stable level. An earlier study reported that A/P measures were better at discriminating faller and nonfaller people in comparison with M/L measures, and A/P measures were particularly associated with fall risk classification [34]. For the aim of this study, we only observed the platform moving around A/P direction. The effects around M/L direction will be observed in our future investigation.

**Figure 2:**
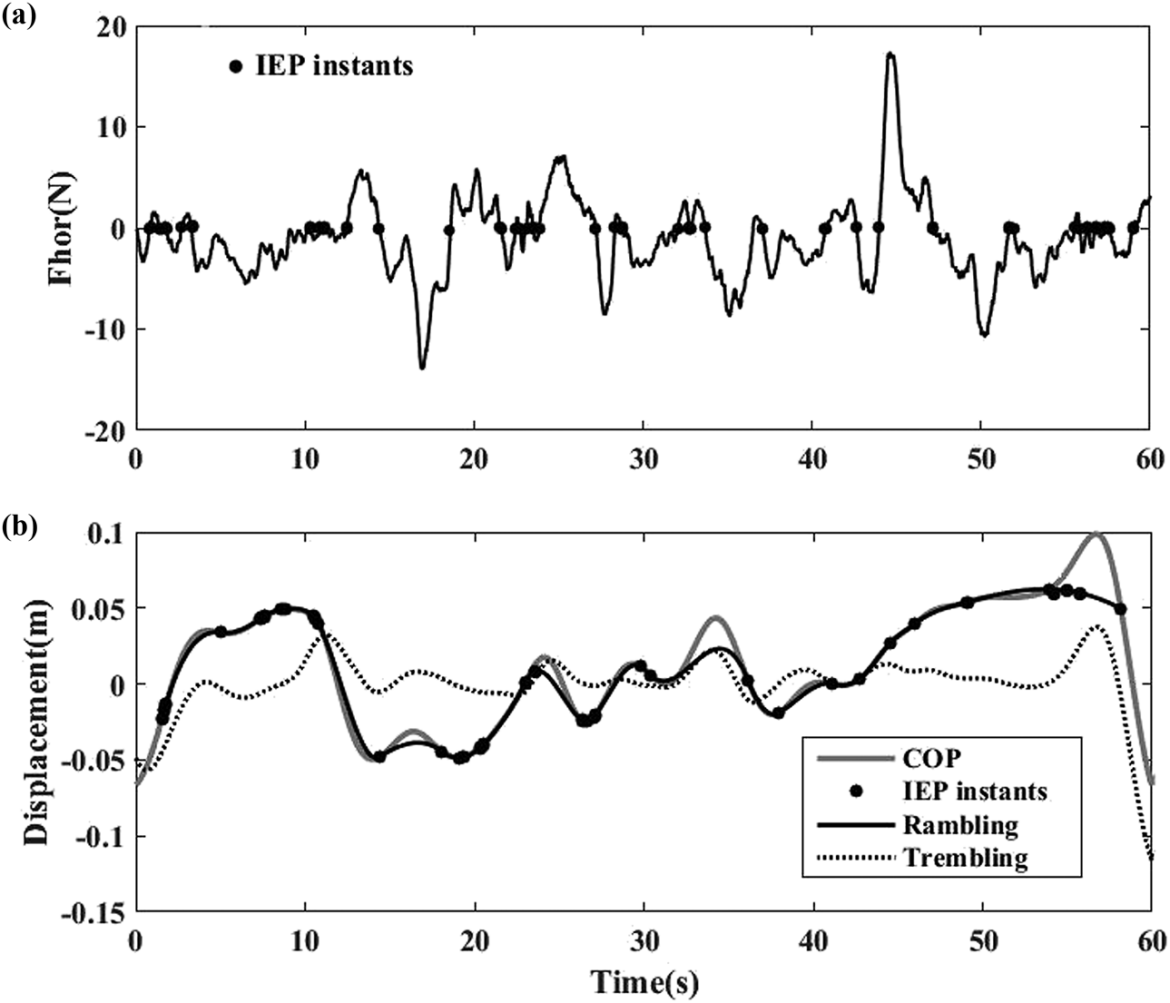
Decomposition of the COP trajectory: (a) trajectory of the IEPs when the horizontal force (Fhor) is zero. (b) Trajectories of COP, RA, and TR. The COP trajectory was recorded when the COP position at the IEP instants (dot marked). The RA trajectory was estimated by the cubic spline interpolation of the COP position at the IEP instants. The TR trajectory was obtained by calculating the difference between COP and RA trajectories. The sample participant is a male, 24 years; height, 1.70 m and weight 62 kg; and A/P direction, 60 s.

During the test, the platform rotated around the A/P direction (*x* direction) with the rotation amplitude at ±4° and frequency at 1 Hz. The movements of COP with the platform were measured under four conditions in a randomized sequence: (1) EO + stimulation off, (2) EO + stimulation on, (3) EC + stimulation off, and (4) EC + stimulation on. During the EO experiments, participants were asked to gaze at a fixed eye-level target approximately 2 m in front of them [35]. The duration of each test was 1 min. To avoid a potential carryover effect between tests, the interval between the tests was 30 min, during which time the participant was asked to leave the platform for relaxing. Experiments under one condition were repeated thrice. The sampling frequency of the pressure transducer was 100 Hz. The data were low-pass filtered with a zero-lag, fourth-order Butterworth filter with a cut-off frequency of 8 Hz (Figure 1b). The parameters include path length, average speed, and average movement range. The basic parameters of COP were measured. We also decomposed these parameters into RA and TR as the COP data alone does not have enough sensitivity and cannot link the measured data with the balance control system. The COP coordinates were decomposed and analyzed for RA and TR in A/P directions using a method described in earlier research [26]. Briefly, when horizontal force (Fhor) was zero [29], we regarded the body as being in equilibrium in that instant and the trajectory of the slow component (RA) was separated from the COP trajectory. Then, we identified the IEPs (defined as COP locations when Fhors was zero; Figure 2a). Subsequently, the trajectory of RA was established by interpolating the discrete IEPs with a cubic spline function; the trajectory of TR was determined by subtracting the RA trajectory from the COP trajectory [26,36]. The IEPs and the trajectories of RA and TR were determined separately from COP anterior–posterior (COP_AP_) and COP medial–lateral (COP_ML_) time series (Figure 2b) [26]. Finally, the following three sway parameters were calculated from the COP time series: length, speed, and range of the trajectory. A reduction of sway parameters was regarded as an improvement in balance. Additionally, in this study, the platform rotated around the A/P direction without rotation around the M/L direction (*y* direction). Hence, the Fhor in the M/L direction could not reach zero, so the Zatsiorsky method could not be used to decompose these parameters into RA and TR. In the present study, we could only use the Zatsiorsky method for parameters in the A/P direction.

### 2.4 Data preprocessing

Experimental data were preprocessed using MATLAB^®^ software (R2017a, The MathWorks Inc., Natick, MA, USA).

Here, three statistical parameters, namely the average range (*R*_*x*_), the average speed (*v*_*x*_), and the length (*L*_*x*_) of the trajectory, were used to assess human balance ability. The relevant equations are as follows:

(1) {R}_{x}=\frac{1}{N}\mathop{\sum }\limits_{i=1}^{N}\sqrt{{{x}_{i}}^{2}},]

(2) {L}_{x}=\mathop{\sum }\limits_{i=1}^{N}\sqrt{{({x}_{i+1}-{x}_{i})}^{2}},]

(3) {v}_{x}=\frac{1}{N}\mathop{\sum }\limits_{i=1}^{N}\sqrt{{({x}_{i+1}-{x}_{i})}^{2}}/\text{Δ}t,]

where *x* is the displacement of COP in the A/P, *N* is the number of data samples, and Δ*t* is 0.01 s. Although from the equations (2) and (3), the index of *v*_*x*_ seems to be a scaled data of *L*_*x*_, yet during our decomposition of speed into RA and TR, the *v*_*x*RA_ and *v*_*x*TR_ may not be scaled with those *L*_*x*RA_ and *L*_*x*TR_. Hence, the index of *v*_*x*_ is an important index in this study.

### 2.5 Statistical analyses

SPSS software (v21.00; IBM, IL, USA) was used for the statistical analysis. A two-way repeated measure analysis of variance followed by Bonferroni *post hoc* correction was used to compare the parameters with the effects of eyes (EO, EC), balance (RA, TR), and stimulation (with vs without dcGVS). Data from at least three independent experiments were analyzed. Final data were presented as the mean ± standard deviation, and *p* < 0.05 was considered statistically significant.

## 3 Results

Tables 1 and 2 show the effect of dcGVS in 0.01 mA on dynamic sway. Table 1 is the data with EC, whereas Table 2 is with EO. The results were analogous in both EC and EO: dcGVS significantly reduced length, average speed, and average range in COP, RA, and TR. The data suggest that the dynamic balance performance was remarkably improved by dcGVS in 0.01 mA in both states (Tables 1 and 2).

**Table 1:** Assessments of the postural sway with EC

		Stimulation		
EC		OFF	On	*p*
Length (m)	COP	1.006 ± 0.045	0.819 ± 0.033	<0.01**
	RA	0.786 ± 0.035	0.636 ± 0.030	<0.01**
	TR	0.828 ± 0.035	0.616 ± 0.028	<0.01**
Average speed (m/s)	COP	0.016 ± 0.001	0.012 ± 0.0005	0.04*
	RA	0.014 ± 0.001	0.009 ± 0.0004	0.03*
	TR	0.010 ± 0.004	0.007 ± 0.0003	0.01*
Average range (m)	COP	0.043 ± 0.001	0.037 ± 0.0011	0.03*
	RA	0.041 ± 0.001	0.035 ± 0.0017	0.03*
	TR	0.019 ± 0.001	0.014 ± 0.001	0.01*

COP, center of pressure; RA, rambling; TR, trembling dcGVS on vs off; *presents *p* < 0.05; and **presents *p* < 0.01.

**Table 2:** Assessments of the postural sway with EO

		Stimulation		
EO		OFF	On	*p*
Length (m)	COP	0.4445 ± 0.0220	0.3740 ± 0.0160	0.01*
	RA	0.4120 ± 0.0310	0.3340 ± 0.0210	0.01*
	TR	0.4004 ± 0.0180	0.3356 ± 0.0180	0.04*
Average speed (m/s)	COP	0.0063 ± 0.0003	0.0050 ± 0.0002	0.01*
	RA	0.0051 ± 0.0003	0.0038 ± 0.0002	0.02*
	TR	0.0046 ± 0.0003	0.0036 ± 0.0001	< 0.01**
Average range (m)	COP	0.0206 ± 0.0015	0.0178 ± 0.0010	0.03*
	RA	0.0239 ± 0.0010	0.0151 ± 0.0011	< 0.01**
	TR	0.0090 ± 0.0009	0.0060 ± 0.0006	0.01*

COP, center of pressure; RA, rambling; TR, trembling dcGVS on vs off; *presents *p* < 0.05; and **presents *p* < 0.01.

**Figure 3:**
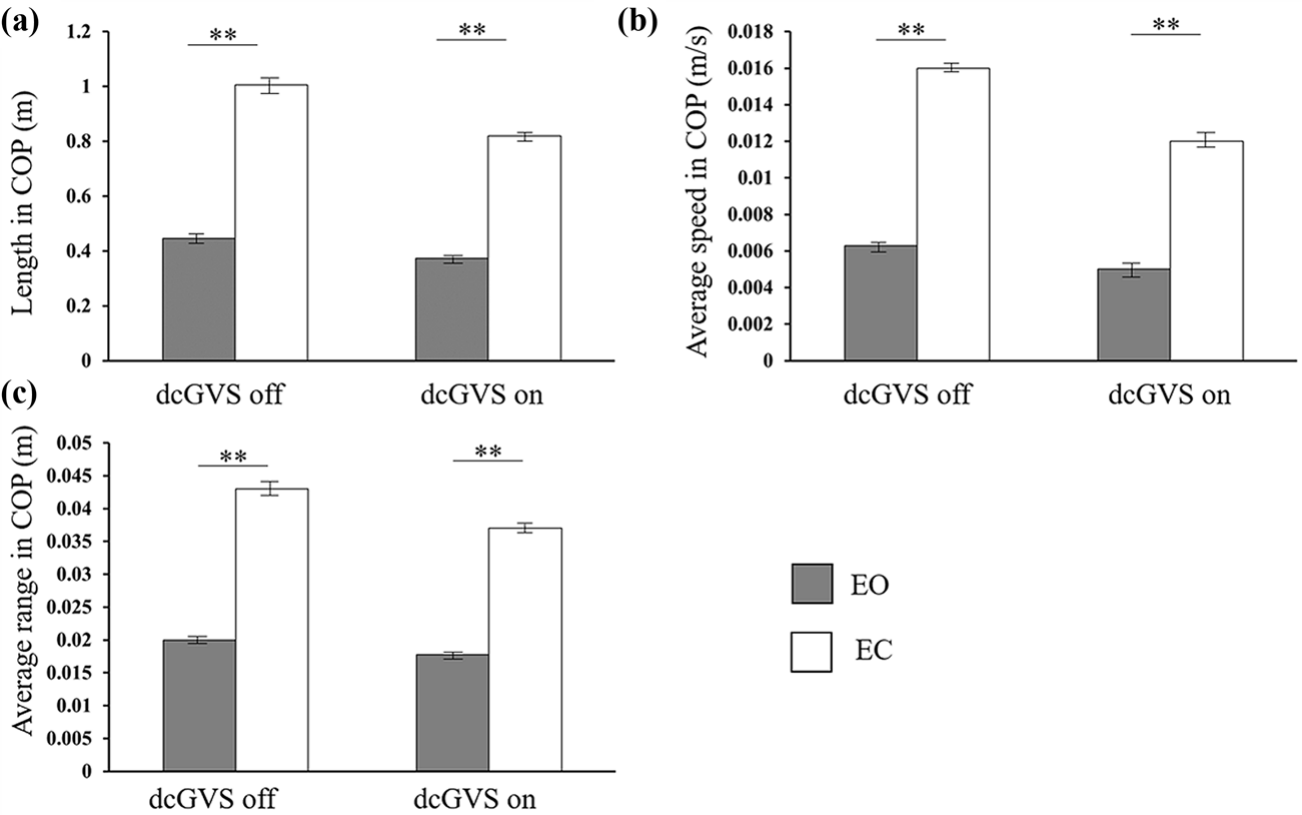
Length, average speed, and average range in COP. (a) Length in COP when dcGVS on and off. (b) Average speed in COP when dcGVS on and off. (c) Average range in COP when dcGVS on and off. All parameters show the same tendency, the parameters in EO were significantly lower than those in EC, indicate EO has a better dynamic balance performance. EO vs EC, ** means *p* < 0.01.

All the sway parameters in COP in EO state were significantly shorter than those in EC state, indicating a better dynamic balance performance when eyes were opened (Figure 3).

**Figure 4:**
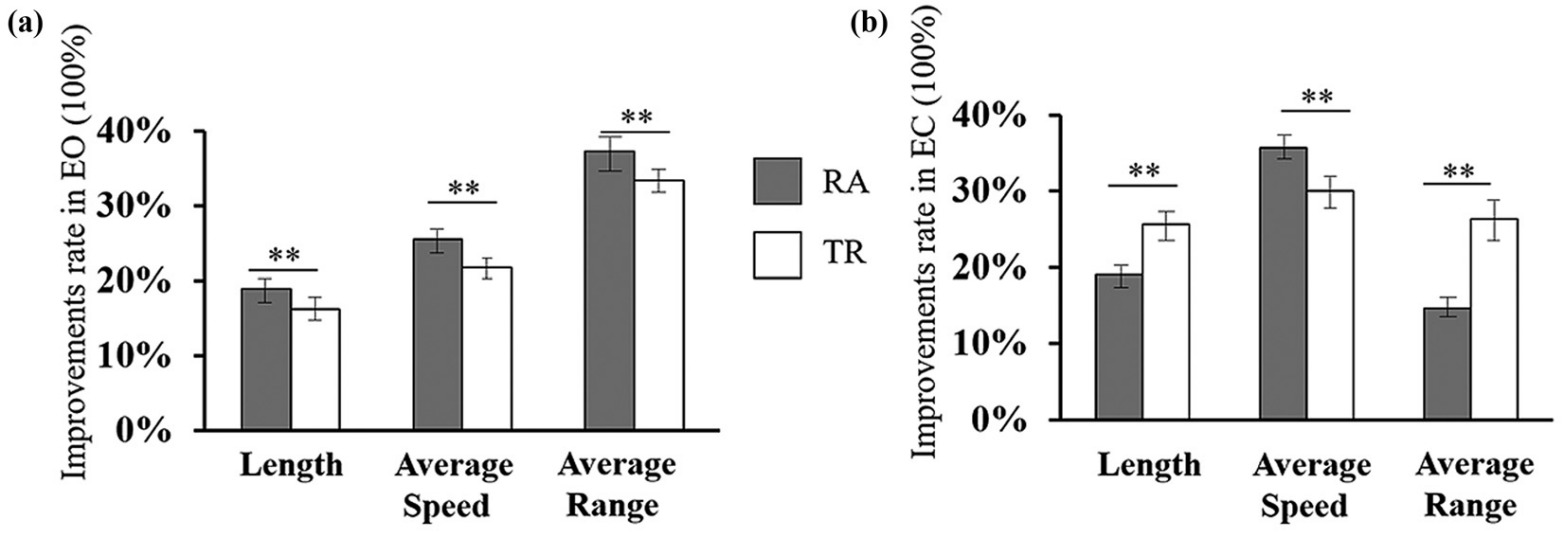
Improvement rates of length, average speed, and average range: (a) Improvement rates by dcGVS when EO and (b) improvement rates by dcGVS when EC. RA vs TR, ** means *p* < 0.01.

With respect to the improvement rate, when eyes were opened the improvement rates of RA trajectory were greater than those of TR trajectory (Figure 4a). But when eyes were closed, the improvement in average speed was higher in RA than in TR, whereas the improvements in length and average range were lower in RA (Figure 4b). Therefore, the changes of “length” and “average range” in EO and EC states presented reversed tendency. These results indicate a different modulation model between EO and EC states, which warrants further investigation.

## 4 Discussion

Using a modified DCRFP system [25], we observed the effects of very low intensity dcGVS on the parameters of dynamic balance in healthy young subjects. We found that this dcGVS significantly improved dynamic balance both in EO and EC without evoking discomfort, strange sensations, or compensatory body sway. Moreover, the analysis of improvement rates between RA and TR exhibited a different trend between EO and EC, indicating a different modulating model in the two conditions. To the best of our knowledge, this is the first report exploring the effects of very low intensity dcGVS (0.01 mA) on the dynamic balance performance and the different balance control modulation between EO and EC. This is beneficial to a deeper understanding of the mechanisms of dynamic balance control.

The most important finding is that dcGVS in 0.01 mA can significantly improve dynamic balance both in EO and EC (Tables 1 and 2). These results are analogous with the findings of earlier studies using nGVS, which enhances both vestibular perception [37] and the vestibular-spinal reflex [37]. It is commonly understood that dcGVS stimulates the vestibular nerves associated with semicircular canals and otolithic organs, and the firing rate of all vestibular afferents is varied with stimulation when a small current is applied via a surface electrode attached to the mastoid behind the ear. When stimulation is on, CNS mistakenly determines “the head is moving” and subsequently triggers a response to “keep balance” [31]. However, conventional dcGVS is used with a larger stimulation intensity (0.5–1.5 mA). Our preliminary studies found when the intensity was more than 0.4 mA, all subjects experienced discomfort. Earlier studies indicated the head and trunk may exhibit compensatory roll when the intensity reached 0.5 mA [38], 1 mA intensity produced sensations of body rotation [39], and a large intensity of 2 mA evoked ocular torsion with both a tonic and a phasic response [40]. Because of this uncomfortable experience with conventional dcGVS, it is used only as a research tool rather than as a treatment. In this study, we found that with stimulation at a relative low intensity (0.01 mA), dcGVS could improve the dynamic balance performance without evoking any discomfort, strange sensations, or compensatory body sway. The detailed mechanisms of this effect remain unknown. We assume that the very low intensity dcGVS might stimulate the vestibular nerves through modulation of the firing rate of all vestibular afferents. Although no noticeable sensory changes were reported by the subjects, the vestibular system may have been “warned” and subsequently made a compensatory response to keep balance, thereby improving the dynamic balance performance. This hypothesis requires further verification.

Balance modulation is complicated and involves several systems, including CNS and the visual, vestibular, and somatosensory systems. When the eyes are opened, visual information is available and can compensate for any failures of other sensory inputs. When the EC, visual input is not available and balance was adjusted mainly according to somatosensory inputs. The results of our study demonstrated a better dynamic balance performance in EO situation (Figure 3), indicating the important role of visual input in the modulation of dynamic balance.

Meanwhile, we decomposed the data of COP and further analyzed the data for RA and TR. It has been known that RA is associated with the complicated response of high CNS, which is an indicator of the comprehensive ability of CNS to obtain an external information to control the body balance, whereas TR is regarded as an indicator of the elementary reflex involved only spinal and muscular, as well as the reaction of the intrinsic mechanical properties of the muscles [26,33,36,41]. Importantly, we found that RA had greater improvement rates in all indices in EO (Figure 4a) situation; however, in EC situation, the results were different. RA only exhibited a greater improvement rate of the average speed but had lower improvement rate in the length and the average range (Figure 4b). The dispersion of improvement rate tendency of the indices between EO and EC suggests a different dynamic balance control modulation depending on whether visual input is on or off. When eyes are opened, visual input is available and RA achieved better improvement, indicating the predominant role of high CNS response. But when EC, visual input is unavailable and the high CNS response was not predominant. Parts of the dynamic balance control had to rely more on the reactions of spinal and muscular elementary reflex. These results are remarkably different from earlier studies in static balance, which reported that RA is approximately three times of TR [26,36]. These results suggest different control models between static balance and dynamic balance. Static balance completely relies on the complicated high CNS reflex, whereas dynamic balance relies on high CNS reflex along with the spinal and muscular elementary reflex. Although our findings also indicated the different modulation models of dynamic balance between EO and EC, the detailed controlling pathway requires further investigation.

Here, we only reported a phenomenon that a very low intensity dcGVS could improve the dynamic balance in young healthy adults. The mechanisms and potential clinical significance remain unknown. It has been reported another stimulation, namely, transcranial direct current stimulation (tDCS) is a promising treatment for neurological and psychiatric disorders. Despite there are great differences between dcGVS and tDCS (stimulation location, current intensity, etc.), whether they share the same mechanisms warrant further investigation in the future.

## 5 Conclusion

The present explorative study used a DCRFP system to investigate the effects of very low intensity dcGVS on dynamic balance in EO and EC. We found that EO exhibited better dynamic balance, and very low intensity dcGVS significantly improved the performance of dynamic balance in both EO and EC without evoking discomfort or any unusual sensations. Moreover, the decomposed analyses found a dispersion of the improvement rate of RA vs TR between EO and EC, which indicate a different dynamic balance control modulation between EO and EC situations. These findings contribute to better understand the mechanisms of dynamic balance modulation. The mechanisms of the effects caused by very low intensity dcGVS require further investigation.
